# Road Risk-Index Analysis Using Satellite Products

**DOI:** 10.3390/s23052751

**Published:** 2023-03-02

**Authors:** Bogdan-Cristian Firuți, Răzvan-Ștefan Păduraru, Cătălin Negru, Alina Petrescu-Niţă, Octavian Bădescu, Florin Pop

**Affiliations:** 1 Faculty of Automatic Control and Computers, University Politehnica of Bucharest, 060042 Bucharest, Romania; 2 Faculty of Applied Sciences, University Politehnica of Bucharest, 060042 Bucharest, Romania; 3 Astronomical Institute of the Romanian Academy, 052034 Bucharest, Romania; 4 National Institute for Research & Development in Informatics—ICI Bucharest, 011555 Bucharest, Romania

**Keywords:** satellite images, risk analysis service, GIS systems

## Abstract

This paper proposes a service called intelligent routing using satellite products (IRUS) that can be used in order to analyze risks to the road infrastructure during bad weather conditions, such as heavy rainfall, storms, or floods. By diminishing movement risk, rescuers can arrive safely at their destination. To analyze these routes, the application uses both data provided by Sentinel satellites from the Copernicus program and meteorological data from local weather stations. Moreover, the application uses algorithms to determine the night driving time. From this analysis we obtain a risk index for each road provided by Google Maps API and then we present the path alongside the risk index in a friendly graphic interface. In order to obtain an accurate risk index, the application analyzes both recent and past data (up to 12 months).

## 1. Introduction

At present, we can make accurate predictions about the weather by using meteorological stations, and can predict earthquakes and even floods [1] by studying flood patterns. Having access to high-speed internet and high-performance smartphones that gather data about the surroundings and knowledge of precise locations using the GPS on these devices helps us to improve our interaction with the environment.

The employees of the General Inspectorate for Emergency Situations often use routes known only by them in order to get to the place of intervention, based on calls to the emergency service [2,3], but sometimes these roads prove to be a higher risk than the alternatives. Even if a road is faster, it does not mean it is safer, so there are a lot of situations when rescuers traveling to the affected areas find themselves near a bridge destroyed by water, a slippery road created by heavy rainfall, or even worse. As stated by the authors of [4], weather plays an important role in the safety of drivers. Every year, a lot of money is invested in creating less risky roads and evolving the infrastructure, but we should not underestimate the forces of nature. Moreover, the authors of [5] explain very clearly the relationship between different weather conditions and accidents and this should make us more aware. Just driving from one point to another without understanding the risks we are exposed to can change our destination to being the hospital.

This is where our service, intelligent routing using satellite products (IRUS), brings a contribution to a safer trip from the source to the destination. It analyzes data from satellite products and finds the safest path between two given points, taking into account multiple data sources.

The main objective of our paper is to eliminate risks when traveling from one point to another by analyzing meteorological data, satellite products, and the duration of the journey. By doing this, we can suggest safer roads for users of the service to take. We calculate the risk of each road using Google Maps API, and then find the one with the lowest risk index.

IRUS is easy to use by anyone, has an intuitive interface, and, as long as it gives a quick answer, people will be willing to use it. The service is different from other existing services because it combines all these data and offers a simple answer by giving the routes alongside their risk indexes.

The scientific relevance of this paper is that it proposes a methodology for calculating the risk for road infrastructure by adopting a data-fusion approach with data from several sources such as satellite images, weather data, and traffic data. The problem of route selection in crisis situations represents an actual and challenging research topic. In the literature, many papers try to solve this problem by proposing different decision-support systems [6,7,8,9,10].

As a current practice in a crisis situation, rescuers generally use a map application such as Google Maps, Waze, or Here Maps. Furthermore, in order to get to their destination, rescuers use these applications in combination with some meteorological data from news channels or from sites in order to be informed about the conditions in the affected areas.

In the following sections we present and analyze the IRUS service components. Section 2 presents the related work by analyzing existing services and solutions. It also provides an analysis of the pre-existing methods and explains why IRUS is considered to bring more value to the actual calculation of a risk index for a given path. The section also describes the need for this application and discusses why we have been motivated to create such a powerful service. We also mention the people that benefit from the usage of this tool, and exlplain how we manage to simplify their tasks. Section 3 describes the workflow of the application and how everything is put together in order to work and give an accurate answer to every request. This chapter also presents the interaction with the application. Moreover, Section 3 presents the actual implementation of the three main services and how they interact with each other. Furthermore, the communication between application components is described in this section and each component is taken apart and analyzed. Section 4 presents how the solution is evaluated and how it behaves. Some metrics are analyzed and concluding results are presented. Finally, Section 5 presents the conclusions of this paper and some ideas for future developments.

## 2. Related Work

In the scientific literature, there are numerous works that present different decision-support systems for crisis management and crisis situations.

In Ref. [6] the authors present the scientific basis of the European Road Safety Decision Support System. The proposed system is based on the following four pillars: taxonomy to identify risk factors and measures and linking them to each other, a repository of studies, synopses summarizing the effects estimated in the literature for each risk factor and measure, and an economic efficiency evaluation instrument (E3 calculator). This system represents a valuable tool, and it can be used for preliminary analyses with respect to crisis situations.

The authors of [7] propose a meta model for specific road crisis situations. The main idea is to set up a collaborative network of heterogeneous actors to solve a critical situation. Thus the proposed meta-model is based on a mediation information system as defined to support the crisis response. We conclude that this approach relies heavily on citizen science and human collaboration, and cannot be applied in a crisis situation that requires rescuers’ intervention and special driving conditions.

In Ref. [8] a decision-support system for safety policy makers is proposed with the aim of being used in road-safety planning, based on the efficiency of previously implemented safety measures. As we can see, this system is not suitable for rescuers, being designed mostly for policy makers.

IRUS comes in handy in this situation because it can fuse the meteorological information with satellite images for road conditions. Our service can evaluate the road by analyzing satellite images and by discovering the flooded portions of the paths. As stated by the authors of [11], we can create models that present the exact evolution of water in different areas and this makes us understand how water progresses.

By using the most recent data (a combination of six-month-old and one-year-old data), IRUS calculates a more accurate risk index for each track and makes the right suggestions in order to keep the rescuers safe until they reach their destination. Moreover, the service takes into account accurate sunrise and sunset times in order to calculate the percentage of road driven during night conditions. By gathering all these data and presenting them in an intuitive form, IRUS becomes easier to use and a trusted source of information.

Our solution can be used by anyone interested in finding out the risk to which he is exposed while driving from a given location to another one, but the most interested persons will be the employees of the General Inspectorate for Emergency Situations. It can also be used by travelers, or by bikers that enjoy going on long rides in nature. By using our service, they will be able to predict accidents happening, and will avoid some risky paths. IRUS has many use cases, and can it be easily used by anyone who is interested.

## 3. Proposed Solution

As mentioned earlier, one of the main advantages of using IRUS is that anyone can use it because of its simplicity and ease of understanding. The graphical interface is friendly enough to offer a pleasant interaction with the service and it helps the user by creating an accessible environment. By just introducing the source and the destination, the user finds the safest route according to IRUS, and he is presented with the directions. Furthermore, if there are multiple routes, the user is prompted with a map displaying the alternatives to the main route.

In order to keep the service simple, the method of accessing the data and querying the server must be uncomplicated. This is why we chose HTTP requests over any other technology; it is simple to use, simple to program, does not need any persistent connection, the answers are not related one to another, we do not need to store any information about the user, and, most importantly, it is fast. Being fast means that the users do not have to wait enormous amounts of time. They have to introduce the information in order for the service to find the best route, and then they are immediately prompted with a map containing highlighted paths to their destination.

When a request to calculate the risk index of a road reaches the server, the processing starts. The server gathers all the needed data in order to compute a response, and then it calculates the risk index for the given roads and after that, it creates an answer for the client. The client-executed code unpacks the data from the server and creates the map in order to present the data in a human-friendly form that is easier to understand.

### 3.1. IRUS Workflow

The application can be divided into two parts: the client-side executed code and the server-side executed code. Both are important for our service in order to complete the main task, which translates as calculating the risk index of a given road.

**Figure 1:**
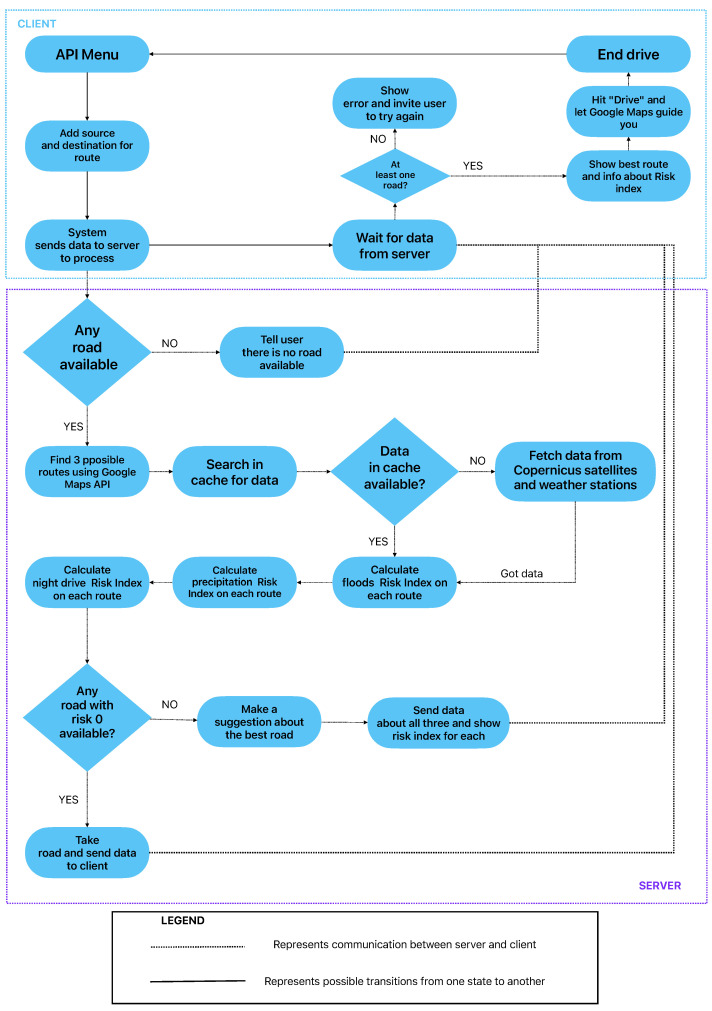
Workflow of the back-end application.

Figure 1 broadly presents how IRUS handles requests coming from the client and the compulsory actions of the service. As it can be observed, the user is prompted with a menu where they introduce the source and the destination. After the client specifies the details of their trip, the client-side code will send an HTTP request to the server with information about the route introduced by the client. After getting the request, the server uses Google Maps API to find out if there are any available routes between the points introduced by the user. In case where no routes are detected, the service displays an error asking the user to check the data they introduced again.

When any roads are available between the points selected by the user, the computation begins, but not before the system checks for a pre-calculated index for the given road. On one hand, by using the caching system we are able to procure an answer faster than doing the computation all over again; on the other hand, all the pre-calculated indexes take up memory. S.V. Nagaraj [12] describes in his book the importance of having a caching system and the different types.

In the case of not finding the data in the cache, the system has to download the needed satellite products in order to complete the request. The service connects to the Copernicus database, obtains the images for processing, and analyzes them by using the SNAP ESA tool in order to produce relevant information for the given road. After the processing, we can calculate the flood-risk index by checking the percentage of the road that was flooded in the resulting image. We will not give the details here because they are described in the following chapters.

After obtaining a flood-risk index, the service will create an HTTP request in order to gather weather information such as temperature, pressure, humidity, and cloudiness. All this data helps IRUS to create a more accurate answer. There are situations in areas where no flood has occurred in the last two or three years, but recently the weather has changed a lot and the area can become flooded in seconds, and the satellite images [13] will still present the road for use as it has a zero risk index. Even though rain is good for agriculture [14], as stated in [15], in large amounts, it can make driving harder. We can monitor the evolution of rain by using satellite images [16], but we cannot create a model or a forecast. This is the case when meteorological information is needed in order to compute a precise risk index.

Furthermore, we add to the calculation of the road-risk index information regarding the time driven during night conditions. This becomes helpful because during the night, people can become easily distracted or even sleepy, and their focus decreases. This data may make people think twice before actually going for a drive. Night conditions imply low visibility, frost in winter, diminished attention, and many other things that people need to worry about. In order to obtain this data, we need to compute the algorithm that is detailed in the following sections of this paper.

After gathering all these data and after calculating the risk index related to the given routes, we send back the answer to the client in order to be processed. The processing on the client side takes the answer and creates an interactive map with the highlighted routes and their information. Alongside the distance and the duration, the risk index calculated for each road is presented.

### 3.2. Implementation Details

IRUS is a complex application divided into two main parts, front-end and back-end processes, and it is composed of multiple services which build up the core of the application. Each of these services is independent of the others and has a well-defined function.

The IRUS tables component serves as the main component of the front end. The user can choose between the following algorithms: sunrise and sunset algorithm at height H [17], civil twilight algorithm [18], and azimuth and altitude [19].

The IRUS tables component contains a selection field of the algorithm whose result the user wants to display. Within this component, the user will pass the following parameters: the latitude, longitude, height, date, and current time of the user. Depending on user choice, the IRUS table1, IRUS table2, and IRUS table3 components are used to query the back-end server to display the results corresponding to each algorithm.

**Figure 2:**
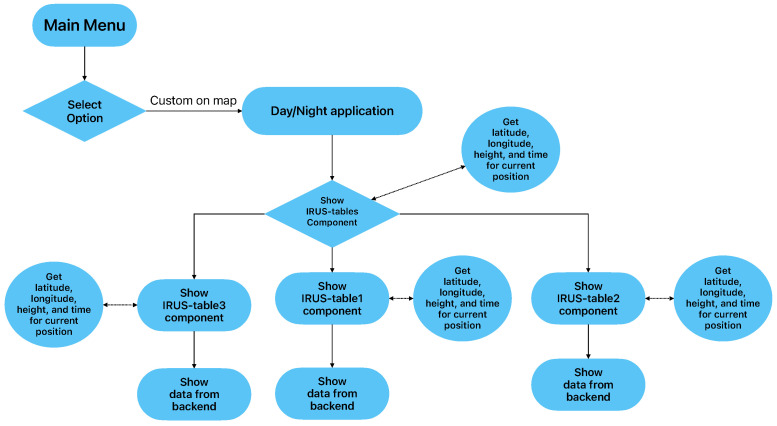
Workflow of the application for day/night menu selection.

Figure 2 presents the workflow of the application for day/night functionality. After the selection, the IRUS tables component will receive the latitude, longitude, height, and time of the current user position. Next, the back-end component will process the parameters and return the results.

**Figure 3:**
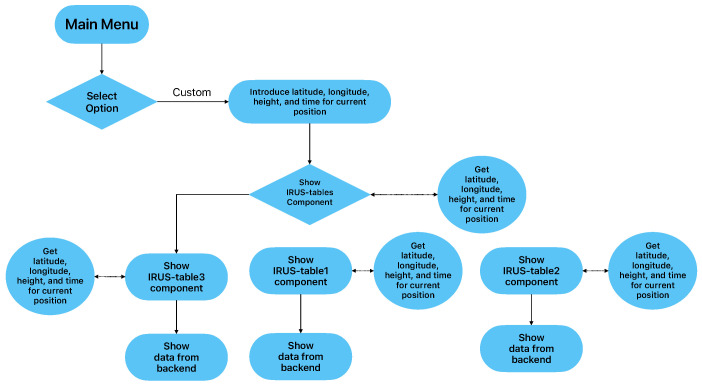
Workflow of the application for custom menu selection.

Figure 3 presents the workflow for a custom menu selection, allowing users to introduce latitude, longitude, date, and time. Next, the IRUS-tables component will receive these parameters and will pass them to the back-end component.

**Figure 4:**
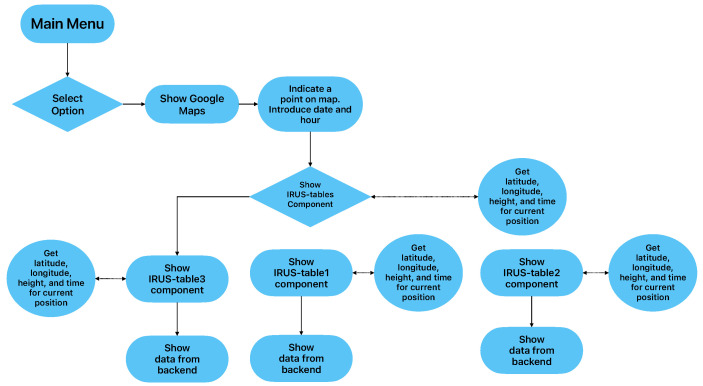
Workflow of the application for generation of routes and information.

Figure 4 presents the workflow of the application for the generation of routes and information. The user has the option to select a point on a map and to introduce the date and the hour. Further, the back-end component receives these parameters, process them, and returns the result.

This paper discusses the back-end side of the project and captures how all these different components communicate in order to solve the problem.

### 3.3. Docker System

Dockers are categorized as PaaS (platform as a service) because they offer an environment for developing applications packed in containers. Furthermore, containers are isolated from one another, and this leads to a safer environment than a local one. By setting up a container, the differences in software or hardware between machines are abstracted, and each one of them ends up with the same environment.

The main reason for large docker container usage is that each service can run isolated from the others in a different container and this brings safety. Whenever a service stops working or is compromised, it can be restarted without affecting others. The second advantage of using docker containers is that they are a lightweight solution for virtual machines. They simply run on top of the software, and do not need a hypervisor. By doing this, they become easier to start and use. Last but not least, the containers create an environment that is similar on each machine, regardless of the operating system or the resources that each machine has. This brings uniformity, and eliminates platform problems such as the right software not being installed, different versions of a tool installed, different operating systems, and so on.

Docker containers allow us to start independent services and isolate them in order to be more secure. This helps us, in the case of a security breach, to let the attacker only access the files inside the container and not those available to the host. A compromised service can be stopped and analyzed and after finding the cause of the problem and fixing it, the service can be restarted. Stopping and restarting a service is much faster than using a virtual machine and this is a huge advantage. The fact that our service will be used by the employees of the General Inspectorate for Emergency Situations makes the service indispensable, and the downtime must be short.

Using dockers brings an enormous advantage because each service used by IRUS for answering the requests is isolated and independent. Each of these services runs separately from the others and if any of them are down, people are still able to ask for information from the others. The technology used in order to start all the services is called **docker-compose** and it uses a script in order to start all the containers. The containers can communicate with each other by using the network which is automatically created on startup.

IRUS is split into three different services that can run in parallel on multiple devices. This is possible to achieve by creating a docker swarm and distributing the requests to any of the machines in the swarm. The most common use case is by using virtual machines from Amazon AWS (Amazon Web Services). After creating a swarm with all the machines, the docker file can be shared between all of them, and the services can be started. The swarm will work like a network of computers where a request will be routed to any of the nodes using a given policy. The docker-compose file used to start the containers is presented in Listing 1.

**Listing 1.** Docker-compose file which brings up the services

version: '3.1'



services:
  interface:
    container_name: server_interface
    build: ./interface
    ports:
      - "82:82"



  main:
    container_name: server_main
    build: ./main
    ports:
      - "81:81"



  front:
    container_name: server_front
    build: ./front
    ports:
      - "80:80"
    command: npm start
    depends_on:
      - main
      -_~_interface



The **interface** service takes care of the day/night algorithm that is used in order to calculate the percentage of the road that has to be driven during night conditions. In order to do this, the algorithm calculates the sunrise and sunset times and takes as input the coordinates of the location, the height and the actual date.

The **front** service is the front-end component that is presented to the user. Here, the interactions between the user and IRUS take place and also here the resulting map where the possible routes are highlighted is presented alongside their risk index computed by the application. As can be seen in the docker-compose file, the service depends on the other two services to be up and running. Therefore, this component waits until the other two components start in order to present the user with the graphical interface.

The **main** service, as the name suggests, is the one that takes care of all the input given by the user, downloads on the server the satellite products that must be analyzed, creates a request for the weather information, and also creates a request for the day/night times for the given locations.

Now that we have talked about the technology used in order to bring the services up, we need to detail the services. In the next subsections each of the three services is analyzed, alongside its well-defined functionality.

### 3.4. Front-End

The front-end component is not the main purpose of this paper, but we need to remind readers of the importance of this part because it is the one that brings an intuitive interface to the user. In order to simplify the interaction with the services, IRUS uses the **Node JS** technology. This helps us create an attractive graphic menu that can be used by users to access the application and to obtain the needed information. After the interface receives the answer to the request, it presents the user with a map that highlights the routes and displays the risk index of each route. In order to create the map, the front-end interface uses Google Maps API and this is the starting point of our application.

Using Google Maps API, the front-end component is able to calculate the routes between the given points. These routes are shaped as arrays of points that have latitudes and longitudes and this is the information needed by the back-end component in order to calculate the risk index.

### 3.5. Back-End

The back end of the core of IRUS is the main part of our service that is able to receive and process requests. When the users introduce the source and the destination of their trip, Google Maps API is used by the front end to generate points on the map. These points are defined by latitude, longitude, and height.

In order to send the data to the back end, we need to pack all the needed information in a form that the main service can understand. As long as we are using **HTTP requests**, we need a format that can be easily transmitted and that is why we chose **JSON**.

Our service does not need a persistent connection as long as the requests are not related one to another. By not using sessions, we bring simplicity and higher speeds to the application. The components that make up IRUS rely on two of the HTTP request types: GET and POST. We use the GET method in order to bring up the interface to the client and the POST method in order to send the data that needs to be processed to the server.

As we described above, we need to transmit the data generated by Google Maps API. The coordinates and their heights must be received by the server in order to start the processing. JSON stands for JavaScript object notation, and is a lightweight data format that can be easily understood by people. Moreover, it can be processed by computers easier and faster than other data types because it works as a dictionary. These objects are key-value objects that allow the user to find the needed information and bring valuable speeds to the processing. An example of the body of a request can be found in Listing 2.

**Listing 2.** JSON body of a request.

{
  "road0": [ { "lat": 10, "lon": 11 },
             { "lat": 10, "lon": 11.1 } ],
  "road1": [],
  "road2": [],
  "info": [[85, 120, 1000], [], []]
}



JSON is a perfect example of a request that comes from the front-end component. In order to understand the fields, we need to explain what the user could have introduced as input data. The client added a source and a destination for their trip, and the Google Maps API used by the front-end service found only one route composed of two points. This is why “road1” and “road2” do not contain any data. As we can see, both points have latitudes equal to 10, but one of them has a longitude of 11 and the other one has a longitude of 11.1. These points are used by the main service in the process of the satellite images. The process is described in Section 3.7, where more details are provided alongside a starting point for our processing, which is the study named “Interferometric processing of sentinel-1 tops data” [20]. We also have the field named “info”, which is represented by an array. Each component of the array is another array with three values, each of them representing information needed by the day/night algorithm in order to calculate the risk of driving during night conditions. The first value represents the height of the starting point of the route, the second value represents the height of the ending point of our trip, and the third one represents the total driving duration, in seconds, of our track.

We use such a data format in order to keep access to each field as simple as possible. There are some alternatives to JSON, one of them being **XML**, but XML is harder to parse and it takes more time to obtain the needed information. The main reason for using JSON is that it is easier to use and to adapt. If there are any other fields needed, we can simply add another key-value object to the representation.

By using this communication channel with HTTP requests, we make each service more independent and we can access each of them separately. If someone knows the format of the request body for each service, they can use it and access them. For the front-end component, we can simply use the GET method in order to take advantage of its features. The JSON for the main service was described above, and we have already mentioned that it is a service that accepts HTTP requests. Moreover, the day/night component can also be accessed by using HTTP requests. Using the POST method, we can send data to be analyzed and an answer will be returned in the form of a JSON. We provide more information about this algorithm in the following sections.

### 3.6. Main Service

The main service of IRUS handles all the requests that come from the front-end component. The service is written in **Python**, and this simplifies the processing. Python is a scripting language that we use in order to gather all the needed data and to analyze it. It is easier to parse the JSON received from the request and easier to make computations. We use this language to download the satellite products, to call the external tool **SNAP** (SNAP ESA tool), analyze the products, read the result, and compute the risk index. Furthermore, the weather data is brought to the server using HTTP requests and the day/night service is called using HTTP requests. This scripting language brings speed both in development and usage and it as such is a powerful tool.

Before starting, we have to mention that in order for our Python scripts to accept HTTP requests we added the **Sanic** library. Sanic is a library that allows Python to become a web server and accept requests coming from the front-end component.

First of all, we have to describe the big picture. This service receives a request containing a JSON in the body from the front-end component. The JSON looks similar to the one described in Listing 6 and takes care of the parsing. In this key-value dictionary object, we find three possible roads with their latitude-longitude points. This information is the most precious one for the satellite product analysis part and it is described in detail in the next paragraphs. After receiving the points, we try to locate the points on the map in order to download the products that fit them best. Having all these images locally, we can now use the SNAP tool and create the resulting image that we use to calculate the flood risk index. After the processing, we obtain the weather data for each point from the input data in order to find the points with low temperatures, low pressure, high humidity, and high cloudiness. In addition to this information, we request data about sunrise and sunset at the first and last point of the road, and try to find the percentage of the road which will be driven during night conditions. In the end, we pack all this information into a JSON and send back the answer to the front end, which prompts the user with a map alongside all the results we send.

Now that we described what IRUS does in order to answer the requests, we can take each component apart and provide more details about how it works. We can divide this service into three main parts: the satellite product analysis, the weather forecast, and the sunrise/sunset algorithm.

Satellite products are images taken by satellites orbiting Earth or other planets. For our study, only the Earth images are relevant, but in the future maybe this service will be used in order to travel between different planets. The images we use are provided by the Sentinel satellites from the Copernicus program [21] and are available to download using HTTP requests. As stated by the authors of the paper “Gmes sentinel-1 mission” [22], this program is an important program for monitoring lots of natural phenomena. One needs an account on the website of Copernicus Open Access Hub in order to access the images and after that, because IRUS uses Python scripts, we use the **wget** GNU Prokect tool in order to download the images.

Because we receive an array of points for each road, one solution would be to download a satellite product for each point, but this takes a lot of time because of the size of the products. In order to optimize the process, we take the first point from the array, download the related product, and then try to find the other points that are included in that image and take them apart from the others. We repeat the process until there are no points left in the array. This can be easily observed in Listing 3.

In order to compute an accurate risk index, we need to look back in time, not only at the most recent product. This is why IRUS takes into account the latest image, an image that was taken at least six months ago, and an image that was taken at least one year ago. By doing this, we provide a more precise computation and information that is closer to reality.

**Listing 3.** Downloading the satellite images.
**while** array_of_points: *
    # take first point and get the surrounding polygon*
    polygon = download.download_and_analyze(
            array_of_points[0][LAT], 
            array_of_points[0][LON],
            target_index)



    points_not_found = []


*# take each point and check where it belongs***
for** point **in** array_of_points:
    p = (**float**(point[LAT]), **float**(point[LON]))


*    # check if inside analyzed polygon***
    if** check_polygon.check_point_inside(p,
        download.get_polygon(polygon)):
        points_in_image[point] = target_index **
    else**:
        points_not_found.append(point)


*# continue searching for rest of points*
array_of_points = points_not_found
target_index += 1



**Figure 5:**
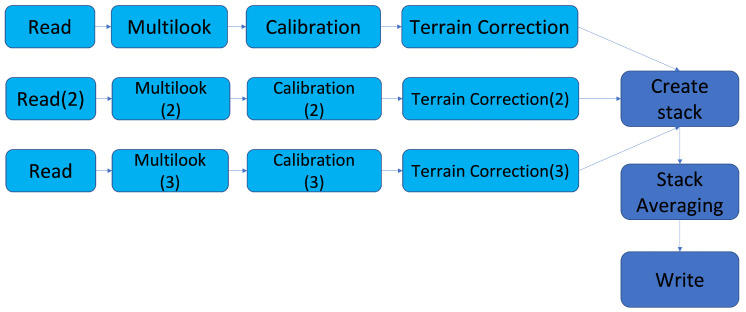
SNAP processing graph.

Now that we have all the needed products, we have to analyze them using the SNAP ESA tool. SNAP is a tool that can handle satellite product analysis and which is able to apply multiple filters on the images. This application is ideal for image processing and can be easily used by our Python scripts. The processing takes multiple filters and transformations to be applied on each triplet of images (the most recent image, the image taken six months ago, and the image taken one year ago). These transformations are presented in Figure 5.

As we can see in the image, there are three identical branches that intersect in a **CreateStack** component. Each branch takes one of the three satellite products and takes care of a part of the analysis. In order to understand what this processing does, we describe each component and its main function.

The **Read** block takes care of the input files. It opens the products and passes them to the next block. The SNAP process needs to open the images and it carries out some background processing in order to present them to the user and in order to allow the user to apply filters or transformations on them. This block should start each processing step.

The **Multilook** block is needed in order to create a product that has a nominal pixel image value. We can create multiple looks by changing the azimuth or/and range resolution cells. This implies improving radiometric resolution but degrading spatial resolution. The resulting image has less noise and approximate square pixel spacing. We use three looks for range and three looks for azimuth, and this creates an image with the mean ground-range pixel size of 30 m.

**Calibration** is essential in each processing step because it radiometrically corrects an image and this leads to the pixel values truly representing the radar backscatter of the reflecting surface. The next component on the chain is the **Terrain-Correction** block and this has enormous importance for our goal. It geocodes the image by correcting the geometric distortions, and the result is a map-projected product. As the name of the component suggests, **CreateStack** overlays the three resulting images and creates a single one. It creates a stack of these three images, but without altering the pixels. In other words, the result is a satellite product that is composed of the three resulting images. Each of them can be accessed separately and all the characteristics are preserved.

The **Stack-Averaging** component is essential for our next step in the process. This block takes the result created by the previous block that contains the three processed images and creates a single image. Each pixel in the resulting image is the average of the three pixels coming from the stacked images. In the next paragraphs, it is described why we need this average and how we use it in our processing.

The last component on our processing graph is the **Write** component that, as the name says, writes the resulting product of our analysis. It is essential to mention here that the type of the resulting image is **GeoTIFF**. We use this format because we want to embed georeferencing information in a TIFF file for easier processing by our Python scripts.

The results of this process which uses SNAP as a primary tool are some GeoTIFF images that contain all our points that build up the routes. Being a GeoTIFF image, each pixel has a latitude and a longitude, so we can use the points provided by the front end in order to obtain a pixel value. By using the GDAL library used in the processing library, Python scripts can easily handle GeoTIFF images and we can access each pixel value using some formulas. First, we need to obtain the coordinates of the corner pixel, because these are embedded in the image. Then we need to find the width and the height of the image in order to calculate the row and the column of the pixel from our latitude and longitude coordinates related to the corner of the image. After that, we can simply access the pixel value matrix and include it in the processing. The retrieval of the values can be observed in Listing 4.

**Listing 4.** Obtain pixel value from matrix.

tif = gdal.Open('target' + **str**(index) + '.tif')
gt = tif.GetGeoTransform()


*# get left -down corner info*
xOrigin = gt[0]
yOrigin = gt[3]
pixelWidth = gt[1]
pixelHeight = gt[5]


*# get pixel matrix*
band = tif.GetRasterBand(1)
data = band.ReadAsArray()


*# calculate pixel offsets from lat/lon*
xOffset = **int**((lon - xOrigin) / pixelWidth)
yOffset = **int**((lat - yOrigin) / pixelHeight)


*# get individual pixel values*
value = data[**int**(yOffset)][**int**(xOffset)]



After numerous processing steps, we conclude that water is present where the pixel value is below 0.05. This means that whenever we take the coordinates of a point and obtain a pixel value that is lower than 0.05, there is a big chance of there being water there. Using this value as a reference and verifying each of the coordinates we have gives us the possibility to calculate how many points have a higher chance of being flooded on the resulting satellite product, and this is how we calculate the flood-risk index.

The component that helps us keep the service fast and which we mentioned at the beginning of this paper is the caching system. Without it, we are not able to have high-speed answers. The processing of a road can take up to an hour or two, depending on how many satellite products are needed. Moreover, depending on the resources of the machine the script is running on, it can take more time to process.

We are proposing a configuration of the docker system that takes up to 14 GB of RAM and 7 CPUs. We are currently using 12 GB of RAM for SNAP processing and most of the cores are at full-percentage usage because SNAP ESA uses, by default, eight or twelve threads for processing, depending on the version. Now that we mentioned the caching system, we have to describe how it is implemented and its benefits. The caching system is dynamic and is kept in memory as a key-value dictionary. For each process in which we use the SNAP tool, the result is an image where each pixel has spatial coordinates. The GeoTIFF image is used by our Python scripts in order to find the exact pixel value for each point in our array of coordinates. Because of the long time of processing, we needed to store the points analyzed in order to quickly answer the following requests.

After SNAP finishes processing, we take the points that build up each route and add them to the key-value dictionary with the related value taken from the resulting image. In this case, the key is the pair of latitude and longitude and the value is the pixel color from the image. The main problem is that the satellite products change at specific intervals and the persistence of our cache should not be too long. Having a cache that holds values for a longer period may lead to inaccurate results because satellite products are updated once every few days. This is why we propose the existence of a background thread which wipes up the dictionary every day at 00:00 AM. By doing so, we maintain a balance between speed and memory.

The following step in our processing is the part where we need information about the weather. This information is available everywhere, and the only thing that we must take into account is obtaining weather data related to a specified position on the map, given by the latitude and longitude coordinates. We are currently using the OpenWeather API for a weather forecast service that offers an API that can be accessed via HTTP requests. This API lets us request weather information for a given latitude and longitude, and the reply contains all the data we need for our processing. Based on the answer, we calculate four risk indexes: low-temperature index, low-pressure index, humidity index, and cloudiness index. All these indexes are calculated based on the same principle that we use for satellite products. We take each point from the array that builds up the route and create an HTTP request in order to receive the weather data for the coordinates of the point. We consider the following as risky threshold values: less than 10 °C for low temperature, less than 1013.25 hPa for low pressure, more than 50% for high humidity, and more than 50% for high cloudiness.

We consider that these values can define a track to be riskier than others because low temperatures can bring frost, low pressure can make people less attentive, high humidity brings slippery roads, and high cloudiness can lead to rain. In order to provide fast answers, we maintain a caching system for this component also. For each coordinate we have, we keep the result of the comparison with the threshold values, resulting in a tuple of four true/false values. This leads to lower waiting times because it is easier to take the value from memory instead of creating an HTTP request. The caching system is implemented in the same way the satellite product caching system is implemented. The main difference is that we can not keep the weather data for a long period. In this case, we propose another background thread that wipes up the dictionary every four hours. The time can be easily adjusted, but if it is longer, the data can become inaccurate, and if it is shorter, the caching system may become useless; these are the two main reasons we choose to use the four-hour threshold. The method used in order to gather weather data is presented in Listing 5.

**Listing 5.** Request data from OpenWeather Api.
*# get info about weather as json*
response = requests.get('https://api.openweathermap.org/data/2.5/' +
		'weather?lat={}&lon={}'.**format**(**float**(lat), **float**(lon)) +
		'&units=metric' +
		'&appid={}'.**format**(key)).json()

*# get temperature, pressure, humidity, cloudiness*
temp = response['main']['temp']
press = response['main']['pressure']
hum = response['main']['humidity']
clouds = response['clouds']['all']



The last component involved in the calculation of the risk index of each road is the service that tells us with precision the sunrise time and the sunset time for a given position on the map, a given hour, and a given height. The service model is detailed in Section 3.7 and here it is detailed how we interact with it and how it helps us to calculate the night-driving risk index. We use HTTP requests for accessing the service and we provide a JSON in the body of the request containing the location, the exact date and hour, and also the height of the point on the map. The service provides us with the sunrise time and the sunset time for the given point. When we started to talk about the main service, we mentioned the existence of a field named **info** in the JSON that the front end sends to the server. This field had three components: the starting location height, the ending location height, and the duration of the route. We use these three components in order to find out the percentage of the road that is driven during night conditions.

We consider the starting time as the time when the request is processed and we add the duration in order to find the arrival time. If the arrival time is the day after the starting time, we tell the user that they are definitely driving during night conditions and that they should take this into account. In case of a trip that falls within a day, we calculate the sunrise time for the starting point and the sunset time for the ending point. After we have this information, we can calculate the number of seconds between the starting time and sunrise and the number of seconds between sunset and ending time. The sum of these seconds represents the portion of the road that implies night driving conditions. With this number and the total amount of seconds to be driven (the duration of the trip, given as an input parameter by the front end), we can calculate the percentage of the road that is traveled at night.

Putting all this information together, we can create a JSON that represents the answer to the request of the user. We can now send the flood-risk index, the weather-risk index, and the night-risk index for each route available, generated by Google Maps API.

Before going into the next section, we must cover an important aspect of the two caching systems. We end up using each of them from two different threads, so we need a method to synchronize them. First of all, we mentioned that we need two background threads that wipe up the key-value dictionaries, one for each of them, and we also have the main thread that carries out all the processing.

The problem is that without exclusive access to the resources, we can end up with a main thread that tries to take data from one of the caching systems, and a related thread to wipe up the data, which leads to data inconsistency.This is why we implemented a synchronization mechanism in our Python scripts that uses **Locks** from the **threading** library. Moreover, the background threads must sleep in the short periods that they do nothing, and we need a mechanism that can announce these two threads when the application stops to stop running. For this mechanism, we choose to use **Events** also from the threading library. This way, the main thread can tell the two threads that they should end their activity if the application closes. By doing so, we eliminate any concurrency issues.

### 3.7. Day/Night Service

We want to give an accurate risk index for each road, and this means that the weather information is not sufficient. The next idea for the calculation of the risk index is to add satellite products in order to analyze the presence of floods on the road. Those two components compute a good risk index, but do not take into account the time of driving. We are humans, and during night conditions our attention diminishes a lot. We become sleepy and tend to relax our muscles, and this is how our eyes start closing. This is one of the most dangerous things that can happen during driving. IRUS is designed to take into account the sunrise and sunset time in order to provide a precise risk index. During the night, roads become riskier than during daylight. In addition to the fact that people tend to fall asleep, the roads may become slippery, the temperature can drop below 10 °C, and the visibility drops significantly, and this results in conditions that should make people think twice before starting their trip.

Taking all this data into account, IRUS provides a risk index that is more accurate than other applications. It uses some simple algorithms that are described in the following paragraphs in order to calculate the percentage of the road that has a higher risk. Both the “Almanac for Computers” [23] and the “Explanatory Supplement to the Astronomical Almanac” [24] present the algorithms used in order to find the time of sunrise and sunset.

Because driving during night conditions means more stops to rest and that people must adapt their speed and driving style to the road conditions, we need to know exactly when sunrise and sunset occur. These two important times for our processing are calculated by the day/night service and are presented to the user in the form of a JSON. The Python scripts are able to calculate the sunrise and the sunset time for a given location described by latitude and longitude, for a given time represented by year, month, and day and for a given altitude. We need the exact date because in each day the sun rises and sets at a different hour. The geographic position is also very important because the sun rises differently regarding the position on the globe. We must not forget the altitude, because the higher it is, the longer the sunset lasts The most common example of the significance of height is the tall buildings from all over the world, where at ground level the sun has already set, while at the top level we can still see the twilight. All this data is given as input to the service using HTTP requests. These requests embed in their bodies a JSON having the structure presented in Listing 6.

**Listing 6.** JSON body of a request.

{
  "date": "2020-03-08",
  "latitude":  22.134423
  "longitude": 44.548672
  "height":    100
}



Now that we know how other services can communicate with the component that calculates the sunrise and sunset times, we can present the algorithm that leads to these times. The algorithm has some important steps that need to be followed in order to obtain a precise result.

First of all, from the given date we have to get to the actual day number of the year. In the following formulas, **n** is the resulting number of the day and $n_{1}$, $n_{2}$, and $n_{3}$ are auxiliary values:

(1) $$n_{1}=floor\left(275*\frac{month}{9}\right)$$

(2) $$n_{2}=floor\left(\frac{month+9}{12}\right)$$

(3) $$n_{3}=\left(1+floor\left(year-4*\frac{floor\left(\frac{year}{4}\right)+2}{3}\right)\right)$$

(4) $$n=n_{1}-\left(n_{2}*n_{3}\right)+day-30$$

The second step is to convert the longitude to an hourly value using the following formula:

(5) $$lngHour=\frac{longitude}{15}$$

For rising and setting times, we calculate the approximate times. In the next formulas we use **RT** for rising time and **ST** for setting time-variable name terminations:

(6) $$tRT=n+\frac{6-lngHour}{24}$$

(7) $$tST=n+\frac{18-lngHour}{24}$$

The third step is to calculate the mean anomaly of the sun, both for rising and setting, with these two formulas:

(8) mRT=(0.9856∗tRT)−3.289

(9) mST=(0.9856∗tST)−3.289

Now that we know the sun’s mean anomaly, we can proceed to calculate the sun’s true longitude for rising and for setting, using:

(10) $$lRT=mRT+\left(1.916*\sin\left(mRT*\frac{\pi }{180}\right)\right)+\left(0.02*\sin\left(2*mRT*\frac{\pi }{180}\right)\right)+282.634$$

(11) $$lST=mST+\left(1.916*\sin\left(mST*\frac{\pi }{180}\right)\right)+\left(0.02*\sin\left(2*mST*\frac{\pi }{180}\right)\right)+282.634$$

Both **lRT** and **lST** may need to be adjusted and brought in the interval [0, 360) by adding or subtracting 360.

The next step in our computation is to calculate the correct ascension of the sun by using the following formulas:

(12) $$raRT=\frac{180}{\pi }*\arctan\left(0.91764*\tan\left(lRT1*\frac{\pi }{180}\right)\right)$$

(13) $$raST=\frac{180}{\pi }*\arctan\left(0.91764*\tan\left(lST1*\frac{\pi }{180}\right)\right)$$

However, right ascension values need to be in the same quadrant as **L** so we calculate the new values. For rise time we use:

(14) $$lqRT=floor\left(\frac{lRT}{90}\right)*90$$

(15) $$rqRT=floor\left(\frac{raRT}{90}\right)*90$$

(16) $$raRTq=raRT+\left(lqRT-rqRT\right)$$

and for setting time we use:

(17) $$lqST=floor\left(\frac{lST}{90}\right)*90$$

(18) $$rqST=floor\left(\frac{raST}{90}\right)*90$$

(19) $$raSTq=raST+\left(lqST-rqST\right)$$

In the following step, we have to transform the right ascension values into hours. This can be performed by following these two formulas, both for sunrise and sunset:

(20) $$raRTh=\frac{raRTq}{15}$$

(21) $$raSTh=\frac{raSTq}{15}$$

Step six comes with the calculation of sun’s declination and this can be performed by using the formulas:

(22) $$sdRT=0.39782*\sin\left(lRT*\frac{\pi }{180}\right)$$

(23) $$cdRT=\cos\left(\arcsin\left(sdRT\right)\right)$$

(24) $$sdST=0.39782*\sin\left(lST*\frac{\pi }{180}\right)$$

(25) $$cdST=\cos\left(\arcsin(sdST)\right)$$

For the following step, we need to calculate the sun’s local hour angle by utilizing the formulas presented below:

(26) $$chRT=\frac{\cos\left(ssh*\frac{\pi }{180}\right)-\left(sdRT*\sin\left(latitude*\frac{\pi }{180}\right)\right)}{cdRT*\cos\left(latitude*\frac{pi}{180}\right)}$$

(27) $$chST=\frac{\cos\left(ssh*\frac{\pi }{180}\right)-\left(sdST*\sin\left(latitude*\frac{\pi }{180}\right)\right)}{cdST*\cos\left(latitude*\frac{\pi }{180}\right)}$$

where **ssh** is the zenith of the sun for sunrise and sunset. For calculating **ssh**, we use these formulas:

(28) $$h_{1}=0.0353*\sqrt{height}$$

(29) $$ssh=90+\frac{50}{60}+h_{1}$$

After that, we can proceed with the calculation of the hour.

(30) $$hRT=360-\left(\arccos\left(chRT\right)*\frac{180}{\pi }\right)$$

(31) $$hRT_{1}=\frac{hRT}{15}$$

(32) $$hST=\arccos\left(chST\right)*\frac{180}{\pi }$$

(33) $$hST_{1}=\frac{hST}{15}$$

The next step implies the calculation of the local mean time of rising or setting, and this can be calculated using the formulas presented below:

(34) $$TRT=hRT_{1}+raRTh-\left(0.06571*tRT\right)-6.622$$

(35) $$TST=hST_{1}+raSTh-\left(0.06571*tST\right)-6.622$$

Now that we have the time, we have to adjust it back to UTC with the following formulas:

(36) UTRT=TRT−lngHour

(37) UTST=TST−lngHour

The obtained time may need adjustments to fit in the interval [0, 24) and this can be carried out by adding or subtracting 24 from the value. The last step in the algorithm implies converting the UTC time to the local time zone of the given coordinates.

Following these steps, we can precisely calculate the time of rising and setting for a given location, a given date, and a given hour. This information helps us to find the percentage of the road that has to be driven during night conditions. The main service uses this data in order to calculate a more precise risk index for each trip, and this is useful both for the employees of the General Inspectorate for Emergency Situations and for normal users that simply want to know how risky is to drive to a given location.

We provide this service as a separate one, because the service may have different use cases. One of them is presented in the previous chapters, but there may be a lot more; imagination is the limit. Furthermore, we developed two other services that can help people calculate the risk index of a road, one that calculates the twilight times (because there are location-based differences of some minutes between sunrise or sunset and twilight)and other that calculates the azimuth and the angle at which the sun hits the car’s windshield. These two services are not currently included in the computation, but are outlined in the following chapters.

## 4. Results

Now that we know the domain where IRUS brings improvements and that we know how it works in order to answer the requests, we have to evaluate the answers. In comparison with other existent services such as weather forecasts, news, and local weather stations, our application brings much more information to the analysis of the risk index of a given road. First of all, we add the satellite product analysis, then we compute weather-risk indexes and finally we use the day/night algorithm in order to find out the percentage of the road to be driven during night conditions. All these information put together gives IRUS a sizeable advantage over the competition.

We analyze the road and offer a more accurate risk index to the users than other applications. This accuracy comes from the fact that we take into account satellite products; not only the most recent one for the given area, but also one satellite images taken six and twelve months ago, respectively. This significantly improves the accuracy of the risk index because we can now know if any area was flooded in the past year and can issue a warning. We analyze the weather information because it is a real-time component that informs us about precipitation, clouds, temperature, and even pressure in different areas transited by the user. Furthermore, the algorithms used in order to determine the exact hour for sunrise and sunset add the human component to the analysis. Humans tend to pay less attention during night conditions and because of the low visibility, more accidents happen. This is why IRUS takes into account that during night conditions, the risk of each road becomes higher and should not be ignored. Knowing how much of your trip you are driving during night conditions should make you aware of the risk that you are exposed to, and should make you think again before leaving the house. By using all the information available, IRUS can deliver a more accurate risk index than the competition.

The downside to using satellite products is the time that each processing step takes. For only one image, the process to receive a result takes around ten minutes. This may lead to hours of processing in the case of longer roads, where we need to process more images. As we described in the first two chapters, the service needs to be fast in order to give fast answers to the users. Even with big amounts of RAM and more cores, the processing can still last for a long time. This is the primary problem of the service and, in order to solve it, we have designed a caching system that holds, for each point that has latitude and longitude coordinates, the value of the related pixel from the resulting image. This allows the service to give an answer a lot faster than at the first use.

**Figure 6:**
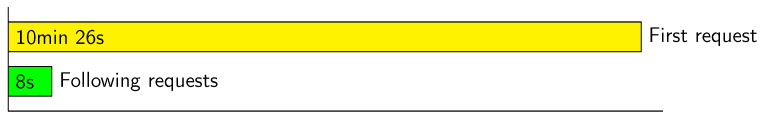
Answer times on first request and next requests.

Figure 6 shows is the time taken at the first request for a road that analyzes only one satellite product and the time taken for the remaining requests.

**Figure 7:**
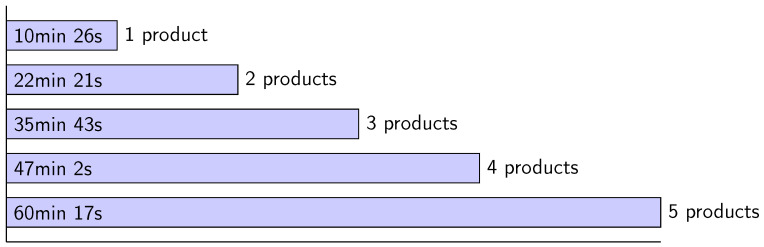
Comparison of answer times for multiple products.

Depending on how many satellite products are analyzed, time can increase significantly. Figure 7 presents the time taken for different numbers of products analyzed. We can observe a linear increase in the answer time for the different number of satellite products.

**Figure 8:**
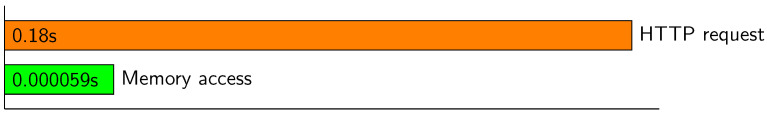
Time difference between HTTP request and memory.

This is why we need the caching system; it helps us in order to give fast answers back to the clients. Furthermore, the caching system is also used for weather data because it is much faster to take the data from a key-value dictionary than to create a request, send it to the **OpenWeather API**, and wait for an answer. This data is changed more frequently than the satellite products and this means that the cache must be flushed every couple of hours in order to maintain an increased accuracy risk computation. Figure 8 presents the difference between a request to the API and a request to the caching system.

We can conclude that IRUS is more accurate than other services, but it also has downsides. Bringing more data to be analyzed in order to obtain a more accurate risk index results in more computation to be carried out and more time spent on gathering data. IRUS can improve these times only if the processing of the satellite images takes less time. This is only possible on a server that has more GB of RAM and more cores than the configuration used and described in the previous chapter. In addition, the analysis of the satellite products can be carried out for a longer period. We can use, for example, products that were created two years ago, one year ago, and the most recent one. This adds value and credibility to the calculated risk index, but the only problem is that these products have to be available for download. As a conclusion, the more data is analyzed, the more accurate the risk index is. In the following chapter we mention some future development cases.

## 5. Conclusions

The unique way that IRUS computes the risk index for each road suggested by the Google Maps API differentiates it from other similar approaches. The service can provide accurate risk indexes, and it takes into account more data sources than other applications.

The fact that lots of accidents happen due to bad weather conditions, such as heavy rainfall, slippery roads, flooded portions of the road, and many others, must help us consider the risks before driving.Road rescuers have the most important job, which involves saving people’s lives whenever they are called. The problem is that before arriving at their destination, these people risk their lives during the ride to get there. Moreover, places that do not have a great infrastructure are the most common for car accidents. In order to allow the rescuers to do their job, we have to keep them safe until they face the real danger. This is why our service tries to do its best in order to calculate a risk index for each trip proposed by Google Maps API in order to offer the rescuers a more accurate overview of the danger they are facing until they reach their destination.

Furthermore, IRUS is designed to be simple to use and it is aimed at not only the rescuers, but also each person that wants to be informed about the risks that their journey may involve. Everyone can create requests by using the front-end interface in order to find out the accurate risks for a given road. This brings more value to our application because it is not designed only for a niche but to fulfill everyone’s requests.

Even though IRUS tries to take into account more than weather forecasts do, we cannot trust the service 100%, because nature is a force that should not be underestimated. It can be unpredictable and we can always be surprised by its power.A good example can be a road suggested by the Google Maps API that was not flooded recently or in the last twelve months, that has a good weather forecast, and that could be driven during daylight, when suddenly a massive rain could start, even with hail falling down from the sky, and the closest river could even swell and flood the road. These are things that cannot be predicted, and this is one of the reasons why we highlight the fact that there are still a lot of data to be taken into account when calculating the risk index of a road.

## Data Availability

Not applicable.

## Abbreviations

The following abbreviations are used in this manuscript:

API	Application programming interface
AWS	Amazon Web Services
ESA	European Space Agency
GDAL	Geospatial Data Abstraction Library
GeoTIFF	Geographical tag image file format
GIS	Geographical information system
GNU	GNU’s Not Unix
GPS	Global positioning system
HTTP	Hypertext transfer protocol
IRUS	Intelligent routing using satellite products
JSON	JavaScript object notation
PaaS	Platform as a service
SNAP	SentiNel application platform
XML	Extensible markup language
